# Physicochemical properties of *M. longissimus dorsi* of Korean native pigs

**DOI:** 10.1186/s40781-018-0163-y

**Published:** 2018-03-29

**Authors:** Gye-Woong Kim, Hack-Youn Kim

**Affiliations:** Department of Animal Resources Science, Kongju National University, Yesan, Chungnam, 32439 Korea

**Keywords:** Korean native pigs, Crossbred breed pig, Meat quality

## Abstract

**Background:**

The meat quality of Korean native pigs (KNP) and crossbred pigs (LYD; Landrace × Yorkshire × Duroc) was examined to generate data useful for selecting native pigs for improved pork production.

**Methods:**

Fifty Korean native pigs (KNP) and 50 crossbred pigs (LYD) were tested. Loin samples (*M. longissimus dorsi*) of the two breeds were analyzed to determine meat quality and sensory properties.

**Result:**

KNP had a higher moisture content than LYD (*p* < 0.05); however, it had significantly lower crude fat and ash content than that of LYD (*p* < 0.001). KNP had significantly higher shear force than LYD (*p* < 0.01). KNP also showed significantly higher cooking loss than LYD (*p* < 0.05). KNP had a lower L^*^ value than LYD (*p* < 0.05); however, it had a markedly higher a^*^ and b^*^ value than LYD (*p* < 0.001). KNP showed significantly higher linoleic acid, linolenic acid, and arachidonic acid content than LYD (*p* < 0.05). Although KNP had significantly better flavor and overall palatability than LYD, it was less tender than LYD (*p* < 0.01).

**Conclusion:**

KNP had a markedly higher a^*^ value than LYD. KNP had significantly higher shear force than LYD. The total unsaturated fatty acid content was higher in KNP than in LYD.

## Background

Pork is sold in seven cuts, the tender loin, loin, shoulder butt, shoulder, leg, belly, and ribs, among which pork belly acceptability in highest compared to the other cuts [1, 2]. Recently, consumers have shown a preferences for high-quality lean meat with low fat content rather than high-fat cuts [3–5]. South Korea’s native pigs are known for their quality meat that fulfills consumer demands [6, 7].

Meat quality is affected by intramuscular fat content, cholesterol, muscular pH, water-holding capacity, drip loss, texture, and cooking loss [8, 9]. Accumulation of intramuscular fat is especially influenced by the specific breed of the pig, types of feeds, and rearing environment, and meat quality heavily depends on intramuscular fat composition; reddish pink meat with little exudation and adequate marbling are considered to indicate high quality and have an important impact on consumer meat choices [10]. Pork quality is affected by breed and feeding, slaughter, and processing. Pig breed has been reported to have a notable impact on meat quality [7, 8, 11]. Korean native pork is a darker and more reddish in color than mat from crossbred pigs; native pork also contains white fat and is tender with high but thin muscle fibers [6, 12]. However, there are no differences in meat color and sensory properties between native and crossbred pigs [13]. Cho et al. [14] reported that Korean native pigs have high levels of marbling and different production yield and meat quality depending on sex and market weight. Additionally, sows have significantly higher L^*^ (lightness) and b^*^ values (yellowness) than boars.

The objective of the present study was to identify factors affecting the quality of Korean native pork by comparing the composition, physicochemical properties, fatty acid composition, and sensory properties of native and crossbred pigs to establish basic data required for developing a continuous production system and for distribution management of Korean native pigs.

## Materials and methods

### Animals and *M. longissimus dorsi* samples

The animals examined in this study were 50 Korean native female pigs (KNP) as well as 50 crossbred pigs (Landrace × Yorkshire ×Duroc; LYD). The two breeds were fed with same feeds, which comply with the National Research Council standard, and were farmed using standard customs (nonghyup feeding standard). Loin samples were taken from the *M. longissimus dorsi* from the 5th to 8th thoracic vertebrae at 24 h postmortem.

### Meat quality and sensory properties

Proximate composition was analyzed as described by the AOAC [15]. Crude protein content was analyzed by the Kjeldahl method, crude fat content by the Soxhlet extraction method, moisture content by ambient pressure drying at 105°C, and crude ash content by dry ashing at 550°C. Shear force was measured with a Warner-Bratzler Shear Meter (Manhattan, KS, USA). Samples were acquired as follows: a raw sample was heated for 30 min in an 80°C constant-temperature water tank and cooled for 30 min. This sample was cut to a thickness approximately 4 × 3 × 2.5 cm, heated and extracted parallel to the grain in a 3 cm diameter core. To calculate cooking loss, a 2 cm thick sample weighting 150 ± 5 g was cut, cooked until its internal core temperature reached 75°C in an 80°C constant-temperature water tank, and cooled for 30 min, after which mass reduction was measured as a percentage. Water holding capacity was measured by centrifugation as described by Laakkonen et al. [16]: a 2-mL filter was first weighed and then weighed again after placing a 0.5 g ground sample in the upper filter of the centrifuge tube. The final pH was measured using a pH meter in the loin core near the ribs on the left side of the carcass at 24 h after slaughter. Lightness (L^*^), redness (a^*^), and yellowness (b^*^) were measured in CIE values using a colorimeter (CR-301, Minolta, Osaka, Japan), which was calibrated against a standard white tile (Y = 92.40, x = 0.3136, y = 0.3196). Fatty acid content was measured as described by Folch et al. [17]. Crude fat in the sample was extracted and melted in 1 mL of chloroform, 100 μL of which was placed in a 20-mL tube. We added 1 mL of methylation agent and incubated the mixture for 40 min in a constant-temperature water tank at 60°C. The final mixture was analyzed by gas chromatography. Sensory evaluation was performed by a panel of 10 trained male and female panelists. The panelists rated the color, juiciness, tenderness, flavor, and palatability of the loin samples for three repeated trials. The panelists evaluated meat quality on a 10 point scale, with one indicating very bad or tough meat and 10 indicating very good or soft meat. The mean values were used for analysis.

### Statistical analysis

The means and standard deviations of the obtained data, including proximate composition and physicochemical properties, were calculated using the SAS program Ver. 3.0 (SAS, Inc., Cary, NC, USA). The mean of the sensory properties was calculated based on the responses of the panel on a 10-point Likert scale ranging from 1 (very bad) to 10 (very good). Statistical significance of the differences among the means was analyzed by the *t*-test and Duncan’s multiple range test.

## Results and discussion

### Proximate composition

The proximate composition of *M. longissimus dorsi* muscle in the KNP and LYD breeds is presented in Table 1. The overall mean moisture content was 73.87%. In terms of breed-specific moisture, the moisture contents of LYD and KNP were 73.67% and 74.06%, respectively, indicating a significantly higher moisture content in KNP (*p* < 0.05). The overall mean of crude fat content was 2.00%. Although the difference was not significant, crude fat content was lower in KNP (1.97%) than in LYD (2.03%). The overall mean of crude protein content was 21.79% with a significant difference between the breeds (*p* < 0.001); crude protein content of KNP (21.45%) was lower than that of LYD (22.13%). The overall mean of crude ash content was 0.69%, with a significantly lower crude ash content in KNP (0.66%) than in LYD (0.72%) (*p* < 0.001). The moisture, fat, and ash content results were similar to those found by Choi et al. [18], while protein content was lower in the present study. The findings of this study were similar to those of a study of crossbred pigs by Jin et al. [3] for moisture (72.19%), protein (22.74%), and fat (3.81%).

**Table 1:** Proximate components of *M. longissimus dorsi* muscles in LYD and KNP breeds

Breeds Items	LYD	KNP	Overall mean	t-values
Moisture (%)	73.67 ± 0.69	74.06 ± 0.18	73.87 ± 0.54	2.12^*^
Crude fat (%)	2.03 ± 0.67	1.97 ± 0.48	2.00 ± 0.57	0.29^NS^
Crude protein (%)	22.13 ± 0.30	21.45 ± 0.60	21.79 ± 0.58	3.91^***^
Crude ash (%)	0.72 ± 0.01	0.66 ± 0.02	0.69 ± 0.03	10.58^***^

All values are the mean ± standard deviation

^*^p < 0.05, ^***^p < 0.001, ^NS^Non-significant

### Physicochemical properties

The cooking loss, pH, and color of *M. longissimus dorsi* muscle in the KNP and LYD breeds is presented in Table 2. The overall mean of cooking loss was 35.05%. KNP showed a higher cooking loss (35.64%) than LYD (34.46%) (*p* < 0.05). This agreed with the results of Jin et al. [3], were cooking loss of KNP crossbreed pork was 36.78%, and similar to the results of Kim et al. [19], where cooking loss of LYD and KNP were 38.66 and 40.56%, respectively. Cho et al. [14] reported that cooking loss was generally higher for pork that had a higher market weight, although the differences were not significant.

**Table 2:** Physicochemical characteristics of *M. longissimus dorsi* muscles in LYD and KNP breeds

Breeds Items	LYD	KNP	Overall mean	t-values
Cooking loss (%)	34.46 ± 1.68	35.64 ± 1.30	35.05 ± 1.60	2.16^*^
pH	5.56 ± 0.10	5.57 ± 0.04	5.57 ± 0.07	0.44^NS^
CIE L^*^(lightness)	53.52 ± 2.47	51.38 ± 2.27	52.45 ± 2.57	−2.47^*^
a^*^ (redness)	6.43 ± 1.30	10.40 ± 2.27	8.41 ± 2.72	5.88^***^
b^*^ (yellowness)	3.27 ± 0.97	4.76 ± 1.31	4.01 ± 1.36	3.54^***^

All values are the mean ± standard deviation

^*^p < 0.05, ^***^p < 0.001, ^NS^Non-significant

The overall mean pH of the two breeds of pork was 5.57, with similar pH values observed both KNP (5.57) and LYD (5.56). This agreed with a study by Park et al. [20], where the pH of pork was similar for all sex and weight groups, and was similar to the results reported by Kang [21].

The mean CIE L^*^ (lightness) value, which represents the brightness of pork, was 52.45 in the present study. The lightness of KNP (51.38) was significantly lower than that of LYD (53.52) (*p* < 0.05). The mean a^*^ value, which represents redness, was 8.41. There was a highly significant difference in the redness of KNP and LYD (*p* < 0.001), with KNP showing significantly higher redness (10.40) than that of LYD (6.43). Similarly, the overall mean b^*^ value (yellowness) was 4.01; however, KNP showed significantly higher yellowness (4.76) than LYD (3.27) (*p* < 0.001). These findings were similar to those of Jin et al. [3], where KNP hybrids showed a significantly lower L^*^ value (46.76) than LYD breeds (three-way cross) (50.55), as well as to the study by Cho [22], where the L^*^, a^*^, and b^*^ values of native pigs were 48.68, 10.83, and 5.53, respectively. Furthermore, our results agreed well with the analysis of native pig properties by Cho et al. [6].

**Fig. 1:**
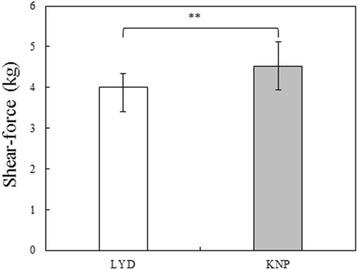
Shear-force of *M. longissimus dorsi* muscles in LYD and KNP breeds. **p < 0.01

**Fig. 2:**
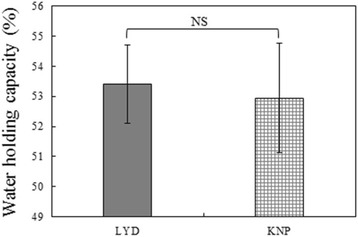
Water holding capacity of *M. longissimus dorsi* muscles in LYD and KNP breeds. ^NS^Non-significant

The shear force of *M. longissimus dorsi* muscle in the KNP and LYD breeds is presented in Fig. 1. The overall mean shear force was 4.26 kg. The shear force of KNP (4.53 kg) was higher than that of LYD (4.00 kg) (*p* < 0.01). Although this was moderately higher than that found by Cho [22] (3.42 kg/in.^2^), this value agreed with those found by Jin et al. [3] and Kim et al. [19], where KNP showed a higher shear force than LYD. The high shear force of Korean native pork may have a large influence on the texture preferred by consumers. The water holding capacity of *M. longissimus dorsi* muscle in the KNP and LYD breeds is presented in Fig. 2. The overall mean water holding capacity was 53.1%. The water-holding capacity of KNP was 52.95%, but this was not significantly different from that of LYD. This was slightly higher than that found by Choi et al. [23], where the water holding capacity of KNP was 42.28%, as well as that reported by Cho [22], where the mean water holding capacity of Korean native sows (weight of 65–75 kg) was 45.81%. The difference may be related to differences in sex and feed.

### Fatty acid composition

Table 3 shows the fatty acid content of *M. longissimus dorsi* muscle in the KNP and LYD breeds KNP and LYD. KNP had a significantly lower composition of saturated fatty acids, such as myristic acid (C_14:0_), palmitic acid (C_16:0_), and stearic acid (C_18:0_), than LYD (*p* < 0.01). Oleic acid (C_18:1n9_), which is an unsaturated fatty acid, showed the highest content, with a significantly higher content in LYD (46.24%) than in KNP (43.87%) (*p* < 0.05). Essential fatty acids, such as linoleic acid (C_18:2n6_), linolenic acid (C_18:3n3_), and arachidonic acid (C_20:4n6_) were significantly higher in KNP than in LYD. The total saturated fatty acid content in KNP was 39.11%, which was significantly lower than that in LYD (41.40%) (*p* < 0.01); in contrast, the total unsaturated fatty acid content was significantly higher in KNP (60.89%) than in LYD (58.60%) (*p* < 0.01). These results were similar to the fatty acid analysis of native pigs performed by Cho [22] and Lee et al. [24], with only the palmitoleic acid content higher in the present study. In addition, our results agreed with those of Kang [21], who reported that KNP had significantly lower contents of myristic acid, palmitic acid, and oleic acid than LYD; however, unsaturated fatty acid (e.g., arachidonic acid) was higher than in LYD. Intramuscular fatty acid influences the flavor of the pork [7], and a high saturated fatty acid is known to help stabilize fat oxidation [25, 26] and meat color [27]. However, unsaturated fatty acid is known as “good” fatty acid because it helps prevent diseases such as arteriosclerosis and hypertension [28, 29], and different fatty acid contents are though to affect the unique flavor of native pork [18, 30].

**Table 3:** Fatty acid composition of *M. longissimus dorsi* muscles in LYD and KNP breeds

Breeds Items	LYD	KNP	Overall mean	t-values
Myristic	1.64 ± 0.13	1.35 ± 0.34	1.50 ± 0.19	6.40^***^
Palmitic	25.30 ± 0.76	24.47 ± 0.69	24.88 ± 0.83	3.14^**^
Palmitoleic	3.10 ± 0.20	3.08 ± 0.28	3.09 ± 0.24	0.23^NS^
Stearic	14.46 ± 1.57	13.29 ± 1.21	13.88 ± 1.50	2.27^**^
Oleic	46.24 ± 2.09	43.87 ± 2.59	45.05 ± 2.60	2.75^*^
Vaccenic	0.26 ± 0.01	0.14 ± 0.14	0.20 ± 0.64	30.16^***^
Linoleic	7.23 ± 0.66	11.77 ± 1.11	9.50 ± 2.48	13.63^***^
g-Linoleic	0.06 ± 0.11	0.05 ± 0.06	0.06 ± 0.09	2.46^*^
Linolenic	0.40 ± 0.32	0.42 ± 0.04	0.41 ± 0.04	2.24^*^
Eicosenoic	0.97 ± 0.07	1.13 ± 0.06	1.05 ± 0.11	6.99^***^
Arachidonic	0.35 ± 0.10	0.41 ± 0.04	0.38 ± 0.08	2.38^*^
SFA^a^	41.40 ± 2.39	39.11 ± 1.88	40.25 ± 2.41	2.91^**^
USFA^b^	58.60 ± 2.39	60.89 ± 1.88	59.75 ± 2.41	2.91^**^
MUFA^c^	50.57 ± 2.28	48.22 ± 2.75	49.40 ± 2.76	2.54^*^
PUFA^d^	8.04 ± 0.72	12.66 ± 1.13	10.35 ± 2.53	13.39^***^
MUFA/SFA	1.23 ± 0.12	1.24 ± 0.13	1.23 ± 0.12	0.24^NS^
PUFA/SFA	0.20 ± 0.02	0.32 ± 0.02	0.26 ± 0.07	14.99^***^

All values are the mean ± standard deviation

^a^*SFA* Saturated fatty acid, ^b^*USFA* Unsaturated fatty acid, ^c^*MUFA* Monounsaturated fatty acid, ^d^*PUFA* Polyunsaturated fatty acid

^*^p < 0.05, ^**^p < 0.01, ^***^p < 0.001, ^NS^Non-significant

### Sensory evaluation

The results of sensory evaluation of *M. longissimus dorsi* muscle in the KNP and LYD breeds is presented in Table 4. The mean visual color was 8.41 points out of 10 points. Although the difference was insignificant, KNP showed a higher score for visual color (8.56 points) than LYD. The overall mean flavor was 8.34 points, with KNP showing a significantly higher evaluation for flavor (8.79 points) than LYD (7.88 points) (*p* < 0.05). The mean tenderness was 8.75 points, and LYD meat was found to be more tender (9.08 points) than KNP meat (8.42 points) (*p* < 0.01). Mean juiciness was 8.44 points, and KNP was found to be significantly less juicy (7.90 points) than LYD (8.98 points) (*p* < 0.001). The mean off-flavor was 8.73 points with no significant difference between KNP and LYD. The overall palatability was moderately high, with a mean of 8.71 points. KNP was found to be significantly more palatable (9.29 points) than LYD (8.13 points) (*p* < 0.001), indicating that consumers had a high preference for KNP meat. These sensory evaluation results agreed with those found by Choi et al. [23] and suggest that KNP was less tender than Duroc or three-way crossbreeds; however, KNP was juicier than the crossbred. Our results did not agree with their report suggesting that KNP had less flavor than three-way crossbreeds, which may be related to differences in the native breed and age at slaughter. Our results regarding tenderness agreed with those reported by Kang [21], where KNP meat was significantly less tender than that of the crossbreds. Furthermore, the overall palatability evaluation was similar to that found by Kim et al. [19], who found that KNP or Berkshire were more palatable than other crossbreds. These results indicate that despite the relatively good evaluations regarding the quality of KNP meat, more attention should be given to weight gain and meat weight increase in breeding management.

**Table 4:** Sensory evaluation of *M. longissimus dorsi* muscles in LYD and KNP breeds

Breeds Items	LYD	KNP	Overall mean	t-values
Visual color	8.25 ± 0.40	8.56 ± 0.56	8.41 ± 0.48	−1.55^NS^
Flavor	7.88 ± 0.86	8.79 ± 0.62	8.34 ± 0.74	−3.00^**^
Tenderness	9.08 ± 0.29	8.42 ± 0.73	8.75 ± 0.51	− 2.92^**^
Juiciness	8.98 ± 0.59	7.90 ± 0.64	8.44 ± 0.62	− 4.26^***^
Off-flavor	8.67 ± 0.50	8.79 ± 0.39	8.73 ± 0.45	− 0.52^NS^
Overall acceptability	8.13 ± 0.68	9.29 ± 0.33	8.71 ± 0.51	− 5.34^***^

Means and standard deviations were denoted by Likert′s scale (10 = very excellent, 1 = very poor)

^**^p < 0.01, ^***^p < 0.001, ^NS^Non-significant (*p* > 0.05)

## Conclusion

Our study analyzed physicochemical properties of *M. longissimus dorsi* of Korean native pigs compared to crossbreed (LYD). KNP had a markedly higher a^*^ value than LYD. KNP had significantly higher shear force than LYD. The total unsaturated fatty acid content was higher in KNP than in LYD. Moreover, KNP which have gained much consumer preference, owing to their relatively bright red color, appropriate texture, and flavor will be a good meat resource.

## Abbreviations

KNP: Korean native pigs
LYD: Crossbred pigs (Landrace × Yorkshire × Duroc)
