# Correction to: Physicochemical properties of *M. longissimus dorsi* of Korean native pigs

**DOI:** 10.1186/s40781-018-0174-8

**Published:** 2018-06-22

**Authors:** Gye-Woong Kim, Hack-Youn Kim

**Affiliations:** Department of Animal Resources Science, Kongju National University, Yesan, Chungnam, 32439 South Korea

## Correction

Upon publication of this article [1], it was noticed that during typesetting, the words ‘Breeds’ and ‘Items’ were accidentally placed next to each other in Tables 1, 2, 3 and 4 whereas they should be discrete. Please see below the correct Tables.

**Table 1:** Proximate components of *M. longissimus dorsi* muscles in LYD and KNP breeds

Items	Breeds		Overall mean	t-values
	LYD	KNP		
Moisture (%)	73.67 ± 0.69	74.06 ± 0.18	73.87 ± 0.54	2.12*
Crude fat (%)	2.03 ± 0.67	1.97 ± 0.48	2.00 ± 0.57	0.29^NS^
Crude protein (%)	22.13 ± 0.30	21.45 ± 0.60	21.79 ± 0.58	3.91***
Crude ash (%)	0.72 ± 0.01	0.66 ± 0.02	0.69 ± 0.03	10.58***

All values are the mean ± standard deviation

* *p* < 0.05, *** *p* < 0.001, ^NS^Non-significant

**Table 2:** Physicochemical characteristics of *M. longissimus dorsi* muscles in LYD and KNP breeds

Items	Breeds		Overall mean	t-values
	LYD	KNP		
Cooking loss (%)	34.46 ± 1.68	35.64 ± 1.30	35.05 ± 1.60	2.16*
pH	5.56 ± 0.10	5.57 ± 0.04	5.57 ± 0.07	0.44^NS^
CIE L* (lightness)	53.52 ± 2.47	51.38 ± 2.27	52.45 ± 2.57	-2.47*
a* (redness)	6.43 ± 1.30	10.40 ± 2.27	8.41 ± 2.72	5.88***
b* (yellowness)	3.27 ± 0.97	4.76 ± 1.31	4.01 ± 1.36	3.54***

All values are the mean ± standard deviation

* *p* < 0.05, *** *p* < 0.001, ^NS^Non-significant

**Table 3:** Fatty acid composition of *M. longissimus dorsi* muscles in LYD and KNP breeds

Items	Breeds		Overall mean	t-values
	LYD	KNP		
Myristic	1.64 ± 0.13	1.35 ± 0.34	1.50 ± 0.19	6.40***
Palmitic	25.30 ± 0.76	24.47 ± 0.69	24.88 ± 0.83	3.14**
Palmitoleic	3.10 ± 0.20	3.08 ± 0.28	3.09 ± 0.24	0.23^NS^
Stearic	14.46 ± 1.57	13.29 ± 1.21	13.88 ± 1.50	2.27**
Oleic	46.24 ± 2.09	43.87 ± 2.59	45.05 ± 2.60	2.75*
Vaccenic	0.26 ± 0.01	0.14 ± 0.14	0.20 ± 0.64	30.16***
Linoleic	7.23 ± 0.66	11.77 ± 1.11	9.50 ± 2.48	13.63***
g-Linoleic	0.06 ± 0.11	0.05 ± 0.06	0.06 ± 0.09	2.46*
Linolenic	0.40 ± 0.32	0.42 ± 0.04	0.41 ± 0.04	2.24*
Eicosenoic	0.97 ± 0.07	1.13 ± 0.06	1.05 ± 0.11	6.99***
Arachidonic	0.35 ± 0.10	0.41 ± 0.04	0.38 ± 0.08	2.38*
SFA^a^	41.40 ± 2.39	39.11 ± 1.88	40.25 ± 2.41	2.91**
USFA^b^	58.60 ± 2.39	60.89 ± 1.88	59.75 ± 2.41	2.91**
MUFA^c^	50.57 ± 2.28	48.22 ± 2.75	49.40 ± 2.76	2.54*
PUFA^d^	8.04 ± 0.72	12.66 ± 1.13	10.35 ± 2.53	13.39***
MUFA/SFA	1.23 ± 0.12	1.24 ± 0.13	1.23 ± 0.12	0.24^NS^
PUFA/SFA	0.20 ± 0.02	0.32 ± 0.02	0.26 ± 0.07	14.99***

All values are the mean ± standard deviation

^a^SFA: Saturated fatty acid, ^b^USFA: Unsaturated fatty acid, ^c^MUFA: Monounsaturated fatty acid, ^d^PUFA: Polyunsaturated fatty acid

* *p* < 0.05, ** *p* < 0.01,*** *p* < 0.001, ^NS^Non-significant

**Table 4:** Sensory evaluation of *M. longissimus dorsi* muscles in LYD and KNP breeds

Items	Breeds		Overall mean	t-values
	LYD	KNP		
Visual color	8.25 ± 0.40	8.56 ± 0.56	8.41 ± 0.48	-1.55^NS^
Flavor	7.88 ± 0.86	8.79 ± 0.62	8.34 ± 0.74	-3.00**
Tenderness	9.08 ± 0.29	8.42 ± 0.73	8.75 ± 0.51	-2.92**
Juiciness	8.98 ± 0.59	7.90 ± 0.64	8.44 ± 0.62	-4.26***
Off-flavor	8.67 ± 0.50	8.79 ± 0.39	8.73 ± 0.45	-0.52^NS^
Overall acceptability	8.13 ± 0.68	9.29 ± 0.33	8.71 ± 0.51	-5.34***

Means and standard deviations were denoted by Likert′s scale (10 = very excellent, 1 = very poor)

** *p* < 0.01, *** *p* < 0.001, ^NS^Non-significant (*p* > 0.05)

The changes to these tables have been actualised by means of this Correction article.

## References

[1] Kim GW, Kim HY (2018). Physicochemical properties of *M. longissimus dorsi* of Korean native pigs. J Anim Sci Technol..

